# Scientific Longevity

**DOI:** 10.1371/journal.pbio.0030409

**Published:** 2005-11-15

**Authors:** Hemai Parthasarathy

## Abstract

This issue of *PLoS Biology* includes the first in a series of "historical" book reviews.

When I was a graduate student, among the most common laments from senior scientists was the lack of scientific scholarship in younger colleagues. If a paper predated or was otherwise excluded from the listing of abstracts in Medline, so they said, it was likely to be at best uncited and at worst recapitulated as an entirely novel discovery. The transition from bound and dusty volumes of printed journals to increasingly backdated electronic archives is, however, progressing (ISI's Web of Science now goes back to 1945), and with it comes the hope of renewed interest in past scientific insights.

Indeed, one promise of open-access publishing is that observations that were out of scientific context and seemingly uninterpretable—thereby relegated to publication in obscure niche journals—will have the opportunity to come together in digital space and inspire synthesis. Another promise is that science educators will have more opportunity to highlight the research process by pointing directly to the source of discovery, rather than solely to the digested contents of textbooks.

We are therefore pleased to present the first in a series of reviews of scientific books that have moved into the public domain, digitized and freely accessible, which promote open-access publishing by virtue of longevity. The author whom we have chosen to highlight first is certainly not obscure—and, indeed, his 19th century theories are the source of renewed controversy in America today. However, Charles Darwin wrote more than just *The Origin of Species*, and as described by the evolution scholar Niles Eldredge in this issue of *PLoS Biology* (DOI: 10.1371/journal.pbio.0030382), it is his earlier unpublished works—the “Red” and “Transmutation” notebooks (1836–1839), the “Sketch” (1842), the “Essay” (1844), and *Natural Selection* (1856–1858)—that help us to trace the development of Darwin's great intellectual achievements.

Darwin's writings have been digitized and made freely available (http://darwinlibrary.amnh.org) by the American Natural History Museum in New York, which will feature an in-depth exhibition on this highly original theoretician, botanist, geologist, and naturalist. The exhibition opens 19 November 2005, and will run until 29 May 2006. It promises to feature live Galápagos tortoises, along with actual fossil specimens collected by Darwin. It will include an elaborate reconstruction of his study at Down House, where he first proposed evolution by natural selection. In short, the organizers hope to bring alive for the public the science and thinking behind a theory that scientists embrace as the most powerful unifying force in modern biological thought. We hope that by highlighting his original words, *PLoS Biology* will foster a deeper interest in and understanding of the origins of evolutionary biology.

More generally, we hope these historical reviews will encourage readers to explore science at its origins. Unlike the contemporary scientific literature—much of which is filled with jargon and acronyms, articulated in the “least publishable unit,” and lacking in elegant prose—the science of the past was often first disseminated in book form, intended to reach contemporary scientific colleagues and Renaissance men alike. Although foci shift and terminology changes, the fundamental approach to scientific problems changes much less quickly. We hope that the words of our greatest thinkers of the past inspire you to address the challenges of the future. [Fig pbio-0030409-g001]

**Figure pbio-0030409-g001:**
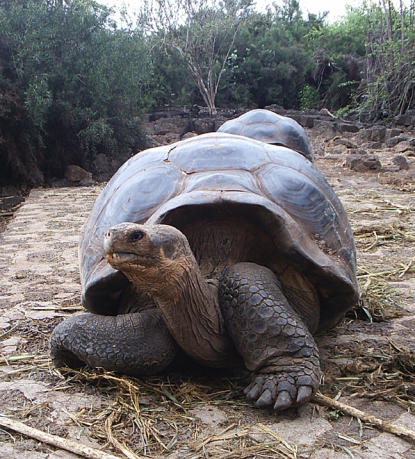
The long-lived Galápagos tortoise (Image: Catriona MacCallum)

